# Mechanisms of long-term cognitive dysfunction of sepsis: from blood-borne leukocytes to glial cells

**DOI:** 10.1186/s40635-015-0066-x

**Published:** 2015-10-29

**Authors:** Monique Michels, Amanda V. Steckert, João Quevedo, Tatiana Barichello, Felipe Dal-Pizzol

**Affiliations:** Laboratory of Experimental Pathophysiology, Graduate Program in Health Sciences, Health Sciences Unit, University of Southern Santa Catarina, Criciúma, SC 88806-000 Brazil; Laboratory of Neurosciences, Graduate Program in Health Sciences, Health Sciences Unit, University of Southern Santa Catarina, Criciúma, SC Brazil; Center for Translational Psychiatry, Department of Psychiatry and Behavioral Sciences, Medical School, The University of Texas at Houston, Houston, TX USA

**Keywords:** Sepsis, Glial cells, Blood-borne leukocytes, Neuroinflammation, Long-term cognitive dysfunction

## Abstract

Several mechanisms are associated with brain dysfunction during sepsis; one of the most important are activation of microglia and astrocytes. Activation of glial cells induces changes in permeability of the blood-brain barrier, secretion of inflammatory cytokines, and these alterations could induce neuronal dysfunction. Furthermore, blood-borne leukocytes can also reach the brain and participate in inflammatory response. Mechanisms involved in sepsis-associated brain dysfunction were revised here, focusing in neuroinflammation and involvement of blood-borne leukocytes and glial cells in this process.

## Review

### Introduction

Sepsis is referred as a systemic inflammatory response due to an infection [1] and presents a wide spectrum of severity: from severe sepsis to septic shock and multi-organ dysfunction syndrome [2]. It is a major cause of death in intensive care unit (ICU) and its incidence is increasing worldwide [3–5]. Sepsis-associated encephalopathy (SAE) is associated with an increased rate of morbidity and mortality. It is not fully understood the exact mechanism that drives brain dysfunction during sepsis development, but brain inflammation and oxidative stress are possible players [6].

**Fig. 1:**
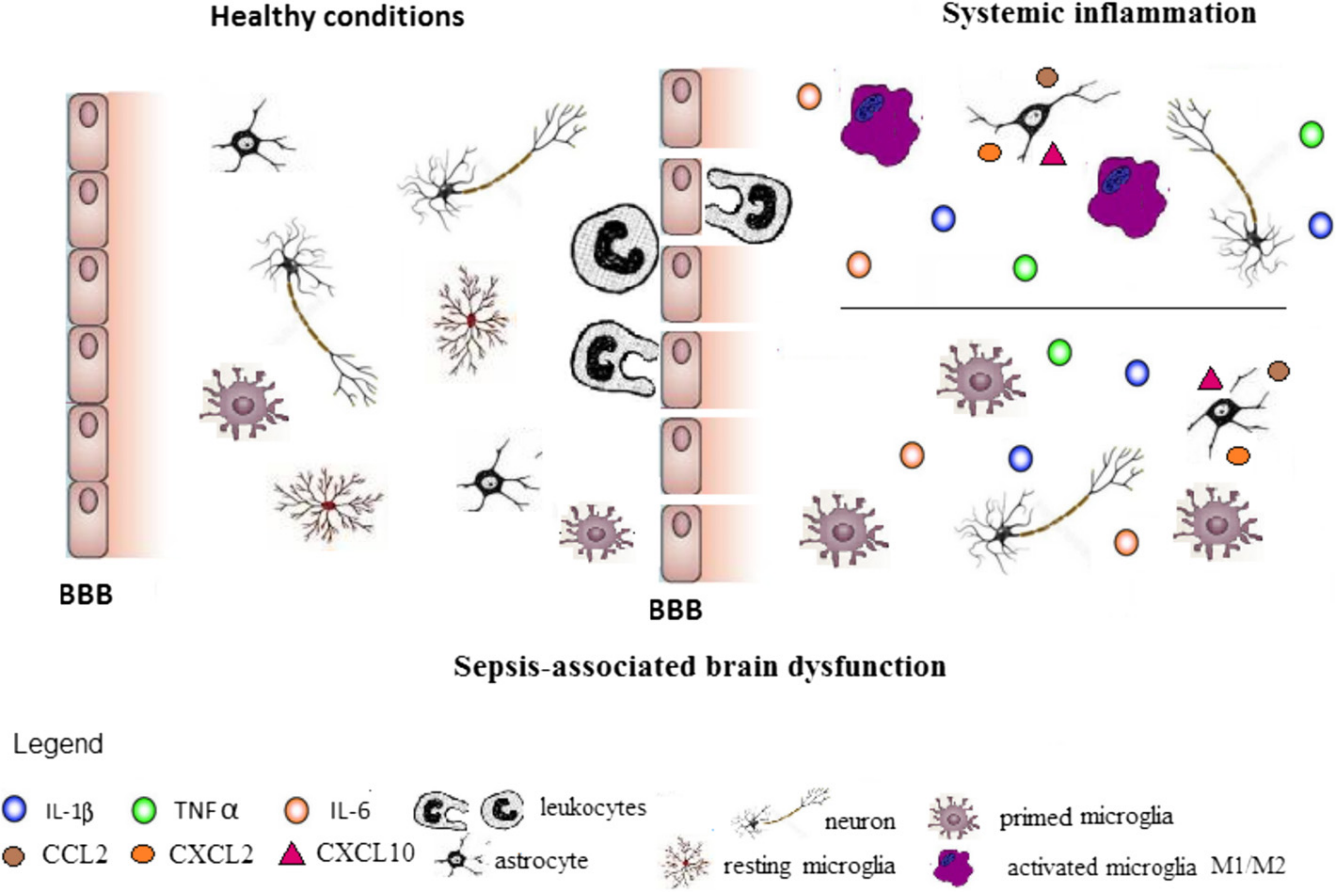
The transition from healthy to sepsis-associated brain dysfunction. In healthy conditions, resting microglia and astrocytes are predominant and are important to maintain several different brain functions. In addition, microglia could be primed, and in this context produces exaggerated levels of inflammatory cytokines in response to a stimulus. Systemic inflammation could activate both resting and primed microglia. Activated microglia could simultaneously exhibit M1 and M2 phenotypes. Despite the fact that classically M1 phenotype is associated with brain damage, during some conditions, the M2 phenotype could also be detrimental to brain function. In addition, astrocytes are also activated, lose the ability to maintain low the extracellular levels of glutamate, to maintain the integrity of the blood-brain barrier, and secrete chemokines. All these modifications are powerful stimuli to the chemotaxis of blood-borne leukocytes that also contribute to sepsis-associated brain dysfunction

Dysfunction of both innate and adaptive immune response plays a role in the induction of abnormal levels of immunoregulatory molecules that result in sepsis [7]. However, participation of effector immune system cells could also impair host response to the infective agents and induces tissue damage [8]. Thus, both an overwhelming inflammatory response and secondary tissue damage and cell dysfunction [9], and a decrease in the immune responses and inadequate infection control [8], could be associated with sepsis pathogenesis [9].

The clinical manifestations of sepsis are variable, depending on the initial site of infection, pathogenic organism, underlying health status of the patient, and time of treatment [10]. The majority of patients with sepsis develop reversible brain dysfunction, called sepsis-associated delirium or septic encephalopathy (SAE) [11, 12]. Moreover, septic patients are at risk for long-term cognitive impairment [12] that could be associated with cerebral atrophy [13–15]. Diagnosing brain dysfunction during sepsis implies a systematic approach of all potential factors. Furthermore, several mechanisms have been proposed to explain the pathophysiology of SAE [6, 7, 16–18]. SAE development involves cellular damage, mitochondrial and endothelial dysfunction, neurotransmission, and calcium homeostasis disturbances. Cerebral blood flow, integrity of blood-brain barrier (BBB) and cerebral water content may also be affected [6, 13, 19]. Apart from brain specific aspects, development of SAE could involve hypoxemia, hypotension, and electrolytes disturbances [20, 21]. Additionally, the long-term effects of sepsis in humans have been evaluated in some studies, all of which underscore the connection between SAE and subsequent cognitive decline [14, 15]. Mechanisms that link SAE and long-term cognitive dysfunction are not well understood, thus evidences that link neuroinflammation and both acute and long-term sepsis-associated brain dysfunction were here reviewed.

### Neuroinflammation

In central nervous system (CNS) immune response to injury is initiated mainly by microglia and astroglia. Neuroinflammation is characterized by activation of microglial cells, followed by changes in permeability of BBB and infiltration of peripheral immune cells into CNS parenchyma. These alterations lead to secretion of inflammatory cytokines and neuronal dysfunction [22], and seem to be a common feature to all neuroinflammatory syndromes [23].

Besides acute symptoms, CNS dysfunction secondary to sepsis is characterized by long-term cognitive impairment. In this context, it has been demonstrated that cytokine levels, oxidative stress, and energetic metabolism alterations seen early after sepsis may persist for up to 30 days and this could be associated with cognitive damage [24–26]. Actually, several groups using different models of sepsis could reproduce cognitive impairment (from inhibitory avoidance to object recognition) that is similar to what is observed in humans [14, 27]. Thus, systemic inflammation is emerging as a significant driver of cognitive decline in the aged and vulnerable brain. A growing body of clinical and preclinical evidence demonstrates that various peripheral inflammatory insults can exacerbate CNS inflammation. In addition, sepsis itself is an independent risk factor for dementia [28]. In this context, severe systemic inflammation can produce a new neuropathology or accelerate cognitive decline previously installed [29]. Furthermore, both human and experimental studies suggest that cognitive impairment and dementia are important risk factors for delirium [30].

Among the far-reaching and sustained systemic effects of sepsis are hemodynamic alterations, which cause the changes in cerebral blood flow that are implicated in SAE. Although reduced cerebral blood flow and oxygenation occur in sepsis, they do not appear to drop low enough to threaten neuronal viability or cause electroencephalogram changes at least in preclinical models of SAE [31]. However, even when cerebral blood flow is sufficient to ensure neuronal integrity, slight reductions could still contribute to SAE when higher energy demands are present, as in the case of cognitive processing [31]. In addition, there are some evidences of alterations in the control of brain perfusion both in animal models and humans [32–35]. These alterations in auto regulation of brain perfusion could be associated with inflammation and the pathogenesis of SAE.

Abundant data have suggested adaptive immunity as a key regulator of brain cell renewal, behavior, learning, and memory. Immune deficiency was linked to impaired brain plasticity, and when adaptive immunity was boosted, brain function was restored or even enhanced [36–40]. Thus, neuroinflammation could be associated with brain (dys)function, inhibition of hippocampal neurogenesis, and disruption of cognitive ability [41]. Although this is clearly demonstrated in neurodegenerative disorders, its role in sepsis associated long-term cognitive impairment is less clear.

### Microglia—do different phenotypes have different impact in brain dysfunction?

Microglia activation has a major role in the generation of oxidative damage and inflammation in the brain during several different CNS diseases. As demonstrated to peripheral immune cells, microglia are able to express several pathogens/damage recognition receptors, such as the toll-like receptors (TLRs). TLR4 and TLR2 expressed in microglial cells have been specifically associated with both neuroinflammation and clearance of aggregated proteins [42, 43]. Thus, it is plausible to suppose that microglia activation is a major determinant of SAE and long-term cognitive impairment after sepsis [44].

Morphology and density of microglia is region specific, being more common in the grey area of the CNS [45]. These differences may be associated with functional heterogeneity, but little is known about the nature of this heterogeneity among and within brain regions. Microglia share common features of cells of the myeloid lineage; they have the ability to secrete a plurality of immunomodulatory molecules, which coordinate signals to neighbor and circulating cells [46]. Thus, during inflammation, activated microglia modify the responses of supporting cells through the release of a diversity of factors [47].

Microglia show morphological and functional diversity in the brain, ranging from the ramified, “resting” phenotype associated with tissue surveillance in the healthy brain to amoeboid, cytokine-secreting, and phagocytic phenotypes in disease states [48]. Microglial activation, which plays a central role in neuroinflammation may be regulated by several intercellular interactions involving cell-surface molecules and soluble mediators, such as cytokines, reactive oxygen species (ROS), and neurotransmitters [22]. In healthy brain, microglial cells display a “homeostatic” phenotype, which monitor the surrounding environment [49]. In this phenotype, microglia express surface molecules and secrete soluble factors, which influence astrocytes and neuron function [50], promote the clearance of cellular debris and aggregated proteins [51]. Accordingly, microglia exhibit at least four functional behaviors: surveillance, neuroprotection, phagocytosis, and toxicity [23]. In addition, microglia could be “primed”, and in this context produces exaggerated levels of inflammatory cytokines in response to a stimulus [52]. Thus, it seems that microglia can be a double-edged sword [53].

Recent studies have demonstrated that microglia may simultaneously exhibit M1 and M2 phenotypes [54]. M1 cells can be neurotoxic, probably through the production of cytokines such as IL-6, IL-12, and TNF-α, reactive oxygen species, and deregulated release of glutamate [55]. Activated brain microglia can be neuroprotective by assuming an M2-like phenotype [56–58], a phenomena similar to what occur in macrophages. In response to lipopolysaccharide (LPS), TNF-α and/or IFN-γ, macrophages become classically activated and acquire an M1 phenotype, which express several pro-inflammatory cytokines and enzymes that promote a sustained tissue inflammation. In contrast, alternative activation in response to IL-4, IL-13, glucocorticoids, TGF-β, and/or IL-10, macrophages differentiate into the anti-inflammatory M2 phenotype, which is associated with the resolution of inflammation and tissue repair [59–61]. It seems that modulation of the M2 phenotype could represent a beneficial aspect of brain immune response during inflammatory injury [62–64], but to date, there is no sufficient information regarding M2 phenotype during the development of SAE.

Free radicals are a major causal factor of secondary insults, such as axonal damage or membrane lipid peroxidation, which result in evolving neurological deficits [65, 66]. Reactive nitrogen and oxygen species, as well as cytokines produced by M1-like microglia, such as TNF-α, can directly induce neuronal death [67–70]. Among the most frequently cited proinflammatory and damaging aspects of microglial activation is the production of nitric oxide (NO) via nitric oxide synthase (iNOS) [32]. iNOS expression in response to sepsis has been detected in neuronal and glial cells [71, 72]. In this context, neurons are highly sensitive to the toxic effects of NO [73]. NO produced by activated microglia is able to induce neuronal apoptosis [74] even when activated glial cells are present at relatively low numbers [75, 76]. In a model of systemic endotoxemia, it was observed an early upregulation of iNOS in several different brain regions, predominantly in microglial cells, and is associated with both neuronal and glial cell apoptosis [77]. Furthermore, iNOS activation can be found even late times after sepsis resolution [78]. NO might also include the stimulation of excitotoxicity, because the application of an acute low dose of the NMDA receptor antagonist MK-801 prevents memory deficits in the cecal ligation and puncture rat model [79].

In addition, microglia are able to generate oxidative burst involving the induction of multiple enzymes/complexes including NADPH oxidase and myeloperoxidase (MPO) [71]. Thus, a sort of strong oxidant agents can be produced by activated microglia, including superoxide, nitric oxide, hypochlorous acid, peroxynitrite, and hydroxyl radical that contributes to the progression of brain damage during SAE [80]. On the other hand, microglial can also help in clearance of free radicals (for example, by regulating ceruloplasmin levels), thus preventing free radical-mediated neuronal damage [70].

Activated microglia can also release large amounts of glutamate that can induce neuronal dysfunction [81]. High extracellular glutamate concentrations in the CNS promote excitotoxicity, which has been involved in various pathological conditions, including acute CNS trauma such as brain or axonal injury [82], ischemia [83], and epilepsy [84], as well as in chronic neurodegenerative disorders such as Parkinson’s disease (PD), AD and amyotrophic lateral sclerosis (ALS) [85]. When CNS is injured, glutamate buffering cells, astrocytes, are lost and the damaged site is repopulated by M1-like activated microglia [86]. Although neurons, astrocytes, and homeostatic microglia can release moderate levels of glutamate, M1-like activated microglia can release toxic amounts of glutamate through a mechanism that involves connexin channels and the cystine/glutamate antiporter system [81, 87]. Excessive stimulation of the ionotropic glutamate receptor N-methyl-d-aspartate (NMDA) in neurons promotes the deregulation of calcium influx, which leads to cellular death [88]. Thus, M1 phenotype exacerbates neuronal dysfunction by several different pathways, which include, at least, cytokine and ROS production and glutamate excitotoxicity [81, 88].

Furthermore, the concept of primed microglia is relevant to the understanding of its role in brain dysfunction during sepsis development. A primed microglia is able to make an exaggerated response to a typical stimulus [80]. Using the ME7 model of prion disease, Cunningham and colleagues demonstrated that the primed brain showed exaggerated response to several challenges, including LPS, poly I:C, IL-1β, and TNF-α [89–91]. This exaggerated response is phenotypically seen both in acute and chronic brain dysfunction after a systemic LPS challenge. This paradigm mimics what happens in the clinical setting. Both acute and chronic brain dysfunction is more severe/frequent in the aged or in patients wild mild cognitive impairment or clinical dementia; the clinical equivalent of the primed brain [92]. Little is known about the mechanisms of microglia activation during sepsis development. Our group recently demonstrated in an animal model of sepsis that the activation of microglia is crucial to acute brain inflammation and oxidative damage [44]. In this setting, microglia activation seems to be depend in part to the activation of CD40–CD40 ligand pathway. The activation of CD40 pathway drives microglia activation, brain inflammation, and oxidative damage and blood-brain barrier dysfunction. Using in vivo two-photon imaging in mice, Gyoneva et al., 2014 [93] showed that systemic inflammation affects the baseline morphology and dynamics of microglia 48 h after the initial stimuli. After systemic inflammation, microglia moved their processes at significantly higher mean speeds, which led to longer distances traveled, and this effect seems to be mediated by adenosine receptors.

Microglia activation is not only relevant to the acute phase of brain dysfunction after sepsis. In a model of endotoxemia, it was observed a long-lasting activation of microglia, but not astrocytes, and this was associated with decrease in expression of plasticity related genes and brain neurogenesis [94]. A single injection of LPS is able to induce microglia activation and inflammatory gene transcription, but not neuronal damage, as long as 2 months after the initial stimuli [78]. This was associated with disrupted synaptic structure and long-lasting behavioral deficits.

### Is there a role for astrocytes in brain dysfunction after sepsis?

During CNS injury, astrocytes become reactive, migrate to the damaged site and form glial scar (reactive astrogliosis) [95]. In pathological conditions, a role for reactive astrogliosis as supportive or detrimental to neuronal survival remains undefined [96, 97]. Studies have shown that astrogliosis can have neuroprotective role by preserving bioenergetics [98] and trophic support [99]. In addition, astrocytes can prevent excitotoxicity [100, 101], decrease oxidative stress [102, 103], and apoptosis in neurons [104]. Astrocyte membranes contain numerous neurotransmitter receptors and transporters and can therefore sense and regulate formation, stability, and efficacy of synapses [105], and this was clearly demonstrated in dopaminergic neurons in vitro [106]. Studies suggest that by transforming from a basal to a reactive state, astrocytes neglect their supportive functions, thus rendering neurons vulnerable to neurotoxins, including proinflammatory cytokines and ROS [104].

Astrocytes seem to be also important to the maintenance of the BBB function [107–109]. They are suggested to regulate BBB permeability, water and ion exchange [110–112]. Presence of numerous astrocyte end-feet close to the BBB allows for a rapid regulation of BBB permeability [113]. Several recent studies have suggested a role for vascular factors in SAE-related injury of the brain vascular endothelium, changes in BBB permeability and microcirculatory dysfunction [114–116]. In this context, they can protect against neuroinflammation by invading T cells contributing to the immune privilege of the CNS [117]. Chapouly et al., 2015 [118] showed reactive astrocytes drive blood-brain barrier opening, via production of vascular endothelial growth factor A (VEGFA). In addition, thymidine phosphorylase (TYMP; previously known as endothelial cell growth factor 1, ECGF1) was identified as a second key astrocyte-derived permeability factor, which interacts with VEGFA to induce blood-brain barrier disruption [119]. Both are co-induced by NFκB1 in human astrocytes as a response to interleukin 1 beta (IL-1β), and inactivation of VEGFA in vivo potentiates TYMP induction. Nowadays, the BBB is understood as a complex regulated system. Terms such as neuro- or gliavascular unit (NVU, GVU) describe the strong influence of the microenvironment on the brain endothelium [120]. Neighboring cell types such as astrocytes, pericytes, microglia, or even neurons are known to influence the functionality of BBB in health as well as in disease, which is supported by their physical proximity and consequent small diffusion distances for signaling molecules [120, 121]. Astrocytes can also downregulate microglial activation by secretion of anti-inflammatory substances such as transforming growth factor (TGF) and prostaglandin E2 (PGE2) [122, 123], resembling a M2 phenotype, and may thereby limiting neuroinflammation.

Dysregulation of astroglial glutamate transporters has been implicated in neuroinflammation. Astrocytes are capable of modulating NMDAR activity through glutamate uptake transporters [121]. Dumont et al., 2014 [124] demonstrated the influence of inflammation on the control of glutamate transmission by astrocytes. Excitotoxic neuronal damage resulting from excessive glutamate is frequently associated with impaired handling of extracellular glutamate by astrocytes. Sepsis is associated with impaired glutamatergic transmission in brain, and inhibition of glutamate uptake by astrocytes through mechanisms that can be modulated by intracellular ascorbate [125]. Astrocytes take up DHAA (dehydroascorbic acid) and use it to synthesize ascorbate that is exported in response to increased glutamate concentrations [126].

Hernandes and colleagues (2014) [127] demonstrated that microglia and astrocytes were activated as many as 5 days after sepsis onset in the hippocampus and that this activation was dependent on the presence of functional Nox2. The results presented by Hernandes et al., 2014 [127] provide evidence that Nox2 is the main source of ROS involved in the oxidative damage to the hippocampus in SAE and that Nox2-derived ROS are determining factors for cognitive impairments after sepsis. They show the importance of Nox2-derived ROS as a central mechanism in glial cells activation and identify Nox2 as a potential target for future therapies to prevent SAE. Nox2 has been shown to regulate intracellular ROS levels in microglia and to result in both amplification of proinflammatory cytokines production and priming of microglia to additional stimuli [127]. Nox2 is essential for glial cell activation and emphasize the critical role of oxidative damage and Nox2-derived ROS as central factors contributing to acute and long-term brain dysfunction after sepsis [128].

In vitro experiments have recently shown that the stimulation of astrocytes with ligands for TLRs 2, 4, 5, or 6 enhances the production of ROS, IL-1β, IL-6, glutamate, and TNF-α, thereby favoring neuronal loss [129, 130]. Thus, activated astrocytes could display a neurotoxic behavior similar to that of activated microglia during inflammation of CNS [22]. An important and differential feature of activated astrocytes is the production of chemokines, including CCL2, CCL5, CCL20, CXCL10, CXCL12, CXCL1, CXCL2, and CX3CL1 [131]. These chemokines are involved in the recruitment of microglia, monocytes/macrophages, T cells and dendritic cells (DCs) into the inflamed sites of the CNS, thus favoring the formation of a more complex and long-lasting immune response during neuroinflammation [22]. Recently it was demonstrated that, as for microglia, astrocytes could also be primed [91]. Differently from microglia, primed astrocytes synthetize chemokines (CXCL1 and CCL2), resulting in markedly neutrophil, T cell and monocyte infiltration in the diseased brain. This suggests that primed astrocytes and microglia have different roles in brain inflammation during sepsis. These results point to a mixed constellation of inflammatory cells inducing brain damage during sepsis. There is an early role to activated/primed microglia that could be sustained for long periods of time after sepsis resolution. In addition, there is a second player that depends on the chemo attractive effects of activated astrocytes, and probably also depends on the breakdown of the BBB that are brain infiltration by peripheral inflammatory cells.

### Blood-borne leukocytes and brain dysfunction

Monocyte-macrophage cells have the ability to phagocytize bacteria and interact with their products, resulting in the release of proinflammatory mediators, such as glutamate, free radicals, proteases, cytokines, leukotrienes, and nitric oxide [132–134] that could contribute to brain dysfunction [135, 136]. This can be further aggravated by the dysfunction of the BBB that is implicated in the pathogenesis of SAE [137]. Systemic-derived and brain-derived inflammatory mediators drive changes in the blood-brain barrier and help the influx of inflammatory cells and toxic mediators into the brain. We had previously demonstrated that activation of the brain microvasculature is an early event in an animal model of sepsis. There was an increase in leukocyte rolling and adhesion, as well as, cell migration into the brain that could contribute to brain inflammation after sepsis [138]. Activation of brain endothelial cells seems to be the main target of circulating inflammatory mediators to activate the brain circuits during systemic inflammation [139]. Recently, it was shown that after sepsis there was an increase in brain endothelial levels of CXCL1 and CX3CL1. This was dependent on leukocyte adhesion and purinergic signaling [140]. This signaling triggered microglia traffic to the injured site and could partially explain how systemic and brain inflammation communicates. In addition, endothelial cell activation seems to depend on estrogen signaling. Systemic LPS, TNF, or IFN administration affects endothelial cell function and BBB integrity only in males and reproductively senescent females but was not apparent in young females [141].

In addition, several immune components can actively cross the BBB via specific carrier inflammation during systemic inflammation [142]. Evidence from sepsis models suggests a role for C5a, and cytokines in the pathogenesis of the breakdown of the blood-brain barrier and the subsequent edema [143–146]. Thus, it seems that BBB dysfunction is a major fueling brain inflammation after sepsis. The mechanisms that drive BBB dysfunction are not fully understood, but include at least the activation of MMP-2, MM9, and MMP8 [147, 148]. These proteases are responsible to degrade key proteins that maintain the functionality of the BBB.

In addition to the innate immunity, lymphocytes could also be implicated in brain inflammation after systemic inflammation. In a neonate model of systemic inflammation, an important step of brain inflammation is the influx of the peripheral leukocytes through the choroid plexus. In this model, brain influx of leukocytes is dependent of an early onset of TH17-mediated immunity [149]. Actually, the choroid plexus has been considered as a possible route for cells to transfer to the central nervous system. Systemic inflammation stimulates expression of TLR and increases CSF cytokines and leukocytes levels, probably by affect blood-cerebrospinal fluid barrier (BCSFB) regulation [150]. This could be a double-edge sword, since deregulation of BCSFB was associated with worse signs of systemic inflammatory response syndrome in an animal model [148]. After systemic inflammation, the choroid plexus upregulated genes cluster into families implicated in immune-mediated cascades, in extracellular matrix remodeling, and in facilitating entry of cells into the cerebrospinal fluid, whereas those downregulated participate in maintenance of the barrier function [151, 152]. These findings reinforce the role for BCSFB in recruit blood-borne inflammatory cells, and in the amplification of brain.

## Conclusions

Glial cells are important for the development of brain inflammation (Fig. 1). It seems reasonable to suppose that they play a central role in brain dysfunction during sepsis development. Furthermore, dysfunction of BBB and migration of blood-borne leukocytes take place in brain inflammation as well (Fig. 1). A more in depth understanding of the role of different subtypes of inflammatory cells and the specific role of each one is needed to further increase our understanding on the mechanisms of sepsis-associated brain dysfunction.
